# Case in Which a Number of Needles Were Extracted from Various Parts of the Body, in a Female

**Published:** 1822-11

**Authors:** C. Otto

**Affiliations:** Copenhagen.


Art. III.—*Case in which a Number of Needles were extracted from various parts of the Body, in a Female.* Communicated in a Letter to Dr. Granville, by Dr. C. Otto, of Copenhagen.†

This paper, which first was read before the Medical Society in Copenhagen, is an account of one of the most interesting cases of the modern time. The patient, about whom it treats, is, first, a proof how much it is possible to suffer; and offers, secondly, a form of disease which certainly belongs to one of the rarest. She has long time attracted the whole attention of the public in Copenhagen; and at last the physician, who already is celebrated as a physiologist and author of a paper "De Vita Fœtus," of "Wounds in the Chest," and of "an Ex-

---

† This communication being written in English, we have thought it would add to its interest to leave it with such alterations only as were necessary to make it intelligible.

amination whether we see with two Eyes or one," has published the case. It seems, indeed, almost impossible; and, although the name of the author would be sufficient, he has however, in order to avoid every suspicion, added the names of a great quantity of medical men who have seen the patient, or have been present at the operation. The case is:—

Rachael Hertz, was first, in the month of August, 1807, affected with a violent colic, which, though several times cured, yet in November was replaced by an erysipelas in the face, that was united with fever, watchfulness, anxiety, disquietude, &c. The physician succeeded in chasing this away; but the fever returned several times, and the erysipelas with it. In April, the patient began to lose her flesh and languish. The face was pale; she had disgust at every food; cardialgia; internal and external heat; all the faculties of mind became weak; she fainted several times in the day, and the whole body trembled so that she could neither stand nor sit. Against this form of hysteria every medicine was tried, but, in fourteen months, without the least success: all the symptoms, often joined to epilepsy, continued with all their force.

At the end of April of the following year, the disease showed itself in another form: to the faintness were added spasmi tonici and clonici, which often were joined with delirium, madness, and even tetanus. This lasted unto the 20th of May in the same year, upon which day a vomitus cruentus arose, by which an immense quantity of coagulated blood was thrown up in three or four days; afterwards all the other symptoms, as faintness, sopor, delirium, cough, singultus, continued to torment the patient, without any relief for seven months. All the physicians despaired now of her life.

In January, 1809, the symptoms changed, but not to the better: to the cough and singultus, painful affections of the stomach, ructationes, vomituritio and tormina, with a fixed pain in the left curvature of colon and perpetual cardialgia, and at last an obstinate retentio urinæ, were united. The cause was undoubtedly a cramp in the intestinum rectum and urethra; and the catheter, clysmata, semicupia, fotus aromatici, &c. prescribed, by which the affections of the bowels were lessened; but the ischuria remained without any relief for two years, and it was necessary every night to evacuate the urine by the employment of the catheter, by which the urethra grew so sensible that the pain was excruciating, and often excited convulsions, which forced the physician many times to wait.

In the beginning of May, 1809, the hysteric symptoms lost suddenly much of their violence and frequency, but did not go away entirely. This apparent tranquillity did, however, not last long; for soon every sleep was changed to a sopor, which shortly after grew so profound that no degree of excitation was able to awaken the girl. The sopor, having continued any time, ended always with a violent delirium and madness, which, as well as the convulsive fits, lasted until night. For some time the physician succeeded in putting an end to the sopor, by snuff in the nose, and so making the patient quiet; but she became soon accustomed to this means, and the dreadful cramps returned, notwithstanding, at the wonted times.

This state lasted for nineteen months, (that is, from the 18th of May, 1809, unto the 8th of December of the following year,) with uninterrupted force. All medicines were in vain; the patient seemed insensible against all: she got, for an instance, eighteen grains of opium in six hours, without any effect at all, and sixteen grains of tartarus stibiatus in two hours produced neither vomitus, opening of the bowels, nor the increased secretion of urine nor sweat.

At the end of November, 1810, the patient fell into such a languor that she was not able to stand on her feet, but always remained in her bed. The convulsive fits ceased, but the sopor increased so much that she almost slept the whole day and night. On the 8th of December, 1810, symptoms of fever attacked her, with diminution of all the other symptoms. The physicians avoiding to disturb nature in her beneficent operations, a perfect crisis followed on the ninth day, and the girl seemed suddenly to be quite well. For two years the health was good. In April, 1813, she had the measles; and in July a remittent fever, with spasmodic cough and bloody vomitus. Refrigerants put all that to flight, and she was again quite well unto the 13th of January, 1814; but on this day a large carbunculus appeared on the anterior and middle part of the left thigh, which was cured about the 20th of May, 1814. In two years she was again free from every complaint; but then she was affected of a rheumatic fever, with violent pain in the stomach and left hypochondrium, and vomitus cruentus. This state lasted four months, unto September. Afterwards she was well for three years; but the 8th of January, 1819, a new tragedy began. A colic pain tormented the patient, and in a much higher degree as before: she could neither sleep nor touch the belly without the most excruciating feeling. To this were added a violent fever, singultus, vomitus cruentus, and evacuation of a black and stinking matter through the anus; and in this dreadful state she could bear neither food nor medicines, nor clysmata, nor fomentations of the belly: she grew, therefore, quite exhausted, and the end of her sufferings seemed not far.

A more exact examination of the abdomen being made, a hard tumor, divided into three lobules, was felt under the umbilicus.

The physician, suspecting an abscess, applied a cataplasma emolliens; but the sufferings continued unto the 12th of the same month (February 1819); the extremities grew cold; and Professor Herholdt resolved, as the last means, to make an incision into the tumor,—no matter, but a little blood ran out; but, examining the deepness of the wound, he met with a hard and metallic body, and extracted suddenly a common needle. All the symptoms grew milder, but shortly after they increased again; and a point being observed in the left lumbar region, at the touch of which the most acute pains were excited, an incision was also made there, and another black and oxydated needle was extracted. So the pains were renewed, and so he, now here, now there, cut through and found needles; and so, from the 12th of February, 1819, unto the 10th of August, 1820, two hundred and seventy-three needles were extracted! In an annexed table, the different places and the number of needles on each is enumerated; so from the left lumbar region, 39; from the left breast, 22; from epigastrium, 41, &c. &c.

In the intervals the girl was quiet; but, as soon as the needle was near the skin, the most acute pain, with fever, singultus, and vomitus cruentus, was excited. It was often necessary to cut through muscles, and the abdomen swelled very much before the needle could be indicated. By a sudden fright, a palsy of the extremities was added to the sufferings of the unhappy; but, after the 10th of August, 1820, after which no needle showed itself, all the bad symptoms ceased; and, by the use of nervina and roborantia, warm baths, vesicatoria, linimenta, the feeling by and by returned, and she seemed in short time quite cured.

In the hope, now, that all was over, the author read this case before the Royal Medical Society; but, since May 1821, the girl suffers of the most obstinate retentio urinæ, after having had a sort of diabetes. What end this takes, the author will publish in the future.

*Copenhagen; August 18th,* 1822.

